# Aerobic exercise increases post-exercise exogenous protein oxidation in healthy young males

**DOI:** 10.1371/journal.pone.0225803

**Published:** 2019-11-25

**Authors:** Gerlof A. R. Reckman, Gerjan J. Navis, Wim P. Krijnen, Roel J. Vonk, Harriët Jager-Wittenaar, Cees P. van der Schans

**Affiliations:** 1 Department of Internal Medicine, Division of Nephrology, University of Groningen, University Medical Center Groningen, Groningen, the Netherlands; 2 Research Group Healthy Ageing, Allied Health Care and Nursing, Centre of Expertise Healthy Ageing, Hanze University of Applied Sciences, Groningen, the Netherlands; 3 Department of Cell Biology, University of Groningen, University Medical Center Groningen, Groningen, the Netherlands; 4 Department of Maxillofacial Surgery, University of Groningen, University Medical Center Groningen, Groningen, the Netherlands; 5 Department of Rehabilitation, University of Groningen, University Medical Center Groningen, Groningen, the Netherlands; 6 Department of Health Psychology, University of Groningen, University Medical Center Groningen, Groningen, the Netherlands; Medical University of Vienna, AUSTRIA

## Abstract

The capacity to utilize ingested protein for optimal support of protein synthesis and lean body mass is described within the paradigm of anabolic competence. Protein synthesis can be stimulated by physical exercise, however, it is not known if physical exercise affects post-exercise protein oxidation. Characterization of the driving forces behind protein oxidation, such as exercise, can contribute to improved understanding of whole body protein metabolism. The purpose of this study is to determine the effect of two levels of aerobic exercise intensity on immediate post-exercise exogenous protein oxidation. Sixteen healthy males with a mean (SD) age of 24 (4) years participated. The subjects’ VO_2_-max was estimated with the Åstrand cycling test. Habitual dietary intake was assessed with a three-day food diary. Exogenous protein oxidation was measured by isotope ratio mass spectrometry. These measurements were initiated after the ingestion of a 30 g ^13^C-milk protein test drink that was followed by 330 minutes breath sample collection. On three different days with at least one week in between, exogenous protein oxidation was measured: 1) during rest, 2) after 15 minutes of aerobic exercise at 30% of VO_2_-max (moderate intensity), and 3) after 15 minutes of aerobic exercise at 60% of VO_2_-max (vigorous intensity). After vigorous intensity aerobic exercise, 31.8%±8.0 of the 30 g ^13^C-milk protein was oxidized compared to 26.2%±7.1 during resting condition (*p* = 0.012), and 25.4%±7.6 after moderate intensity aerobic exercise compared to resting (*p* = 0.711). In conclusion, exogenous protein oxidation is increased after vigorous intensity aerobic exercise which could be the result of an increased protein turnover rate.

## Introduction

Whole body protein metabolism consists of protein utilization and protein breakdown that includes protein oxidation. Protein utilization is important for health maintenance and disease recovery considering that loss of body protein results in negative outcomes such as diminished physical function, impaired clinical outcome of disease, and deteriorating mental function [1,2]. Proteins that are degraded into amino acids can be reutilized for protein synthesis or irreversibly eliminated from the body whereby the amino groups are metabolized in the urea cycle and excreted as urea in urine [3]. The remainder of the molecules enter glycolysis or the citric acid cycle whereby both pathways eventually lead to the formation of CO_2_ which is subsequently exhaled [4]. The maintenance of lean body mass relies on both adequate ingestion of dietary protein and also the successive utilization of ingested dietary protein derived amino acids [5]. The paradigm of anabolic competence categorizes the factors related to the ability of lean body mass maintenance into three domains: nutrition, internal milieu, and physical activity [6]. Progressive elucidation of how these domains and their factors, such as protein oxidation, (inter)act will contribute to improved fundamental comprehension of whole body protein metabolism and to further developing current interventions to optimize protein utilization for lean body mass preservation.

Protein synthesis has received considerable attention in research from the molecular to the physiological level [7–11]. Physical activity, i.e., daily activity and exercise, is an important stimulus for post-exercise protein synthesis and thus protein utilization [12–15]. Protein oxidation, on the other hand, has been less well studied based upon the number of available publications. Measuring exogenous protein oxidation non-invasively after the ingestion of naturally enriched ^13^C-milk protein is a compelling addition to fundamental and clinical research on protein metabolism. At rest, the bodily pool of proteins is in a constant state of turnover to ensure renewal and replacement of protein to meet ever-changing demands. Approximately ~250 g of bodily protein per day is degraded and reused for protein synthesis, and ~25 g of protein (~10%) per day is lost as irreversible oxidation [16,17]. Because net protein synthesis occurs post-exercise, exercise is considered to be an anabolic trigger [18,19]. Therefore, it is conceivable that exercise suppresses exogenous protein oxidation post-exercise so that exogenous protein oxidation could be subsequently related to anabolic competence. Determining whether aerobic exercise affects exogenous protein oxidation is important within the clinical setting because, if the anabolic effect of exercise is reflected by a decrease in ^13^C-milk protein oxidation, it will provide the clinician with a relatively simple tool for assessing protein utilization. However, it currently remains unclear to what degree variations in exogenous protein oxidation occur in the first hours immediately after different levels of aerobic exercise.

Therefore, the aim of this study was to determine the effect of two levels of exercise intensity in healthy young men on immediate post-exercise exogenous ^13^C-protein oxidation.

## Materials and methods

### Subjects

Sixteen healthy, young male subjects were recruited via local advertising from February 2017 until June 2018 in the form of posting flyers in the Groningen university buildings. To obtain a homogeneous group of subjects, the following inclusion criteria were applied: aged between 18–30 years, body mass index (BMI) between 20–25 kg/m^2^, being able to fast overnight, being able to speak the Dutch language, and provide written consent. Exclusion criteria were: having a diagnosed disease and/or being medically treated (e.g. diabetes mellitus and heart condition), milk protein allergy or intolerance, smoking, use of drugs, drinking on average more than two glasses of alcohol per day, waist circumference larger than 102 cm [20], and scoring higher than ‘low risk’ on the American College of Sports Medicine checklist for physical exercise [20]. These exclusion criteria were selected in order to minimize possible confounding effects of subject characteristics over the exercise conditions. The study was approved by the local Medical Ethical Committee at the University Medical Center Groningen (NL59615.042.16, METc 2016.585), conducted in accordance with the Helsinki Declaration of 2013, and registered in the Dutch Trial Register under registration number NTR6154. After receiving an information letter regarding the purpose and practical procedures of the study and attending an informative meeting with the researcher, every participant provided his written informed consent prior to participation.

### Study protocol

For each participant, age (years), height (cm), waist circumference (cm), body weight (kg), BMI (kg/m^2^), fat-free mass (FFM, kg), and resting metabolic rate (MetaLyzer 3B, Cortex) were measured. FFM was measured with a bioelectrical impedance analysis (Quadscan 4000, Bodystat Ltd., Isle of Man, British Isles). To determine each subject’s risk of performing physical exercise, the American College of Sports Medicine Risk Classification Calculator was used [20]. Resting heart rate was measured after the participant had been seated for ten minutes. Thereafter, the Åstrand test, i.e., a low risk sub-maximal cycle ergometer test on a stationary bike (Corival, Lode B.V., the Netherlands), was performed to estimate the subject’s VO_2_-max [21]. The latter was required in order to determine the required wattage per individual for each of the exercise conditions which included cycling for 15 minutes with resistance at 30% (moderate intensity) and 60% (vigorous intensity), respectively, of the person’s estimated VO_2_-max. The participants were instructed to maintain a pedalling rate of 70 revolutions per minute (rpm) and were guided by a metronome and the display of the cycle ergometer which displayed live rpm data. The saddle height was tailored and documented for each of them at the beginning of the Åstrand test and was repositioned for each subsequent exercise condition.

Prior to each of a total of three protein oxidation breath tests, the subjects were instructed to keep a three-day food diary with respect to their habitual food intake, to calculate their average daily intake of energy (kcal), protein (g), protein en%, animal protein (g), plant protein (g), carbohydrates (g), carbohydrates en%, fat (g), and fat en%. The calculations on dietary intake were performed with Evry (Evry BV) which uses the NEVO 2013, RIVM database [22]. Also, 24-hour urine was collected on one of the days for which the subjects kept the food diary. From each 24-hour urine sample, urea and creatinine concentrations were measured in order to calculate the individual’s actual protein intake [23].

On the evening prior to the breath test, subjects were instructed to begin fasting overnight (i.e., only consumption of water, tea, or coffee without milk and sugar was allowed) from 22:00 PM onwards and to arrive sober the next morning at 08:30 AM. During each test, three basal breath samples were collected and averaged to establish their baseline ^13^CO_2_:^12^CO_2_ ratio. At 09:15 AM, 30 g of naturally enriched ^13^C-milk protein dissolved in 500 ml water was consumed within five minutes. From 09:25 AM until 14:45 PM (5.5 hours), a breath sample was collected every ten minutes. Within this period, individuals were instructed to remain seated in an upright position and not to eat or drink. They were allowed to work on a laptop, to read, and to write.

For each of them, on three separate days, three breath tests were performed: the first after no exercise, the second after 15 minutes of cycling at moderate intensity, and the third after 15 minutes of cycling at vigorous intensity. The order of exercise conditions, moderate versus vigorous intensity, was randomized. Between the breath tests, there was a washout period of at least one week in order to return to baseline ^13^CO_2_ levels.

### Naturally enriched ^13^C-milk protein as substrate

The ^13^C-milk protein was produced by placing lactating dairy cows on a diet naturally enriched in ^13^C (corn). The resulting milk was measured for several days to determine its ^13^C-enrichment until the ^13^C-enrichment of the milk stabilized (-14.21). Thereafter, the milk was processed by NIZO food research (Ede, the Netherlands) into food grade milk protein powder.

### Sample size analysis

A pilot experiment in which the exogenous protein oxidation was measured in resting condition after the ingestion of 30 g of milk protein. A standard deviation (SD) of 3.37% was estimated at the area under the curve end point (t = 330 minutes) over six repeats. Based on a true difference of 3.00 in means and an SD of 3.37, a desired power of 0.90, significance level 0.05, a two-sided paired t-test sample size calculation revealed that 16 (15.31) subjects were required.

### Calculations and statistical analysis

The exercise conditions included cycling for 15 minutes at 30% (moderate intensity) and 60% (vigorous intensity) of the subject’s VO_2_-max. To convert the estimated VO_2_-max to the corresponding wattage for each cycling condition, the following calculations were made. First, the parameters were calculated for the linear relationship [24,25] between VO_2_-max and heart rate whereby the resting heart rate was assumed to be equal to 3.5 ml of O_2_/kg body weight/minute [26], and maximum heart rate was assumed to be equal to the estimated maximum VO_2_-max (ml of O_2_/kg body weight/minute). Maximum heart rate was estimated with the following formula [27]:

208beatsperminute(bpm)−0.7xage(years)

Second, the linear relationship between heart rate and power output (watts) at submaximal power output was calculated [28] whereby the heart rate at the end of warming up at 50 watts corresponded to 50 watts, and the heart rate at the end of the Åstrand test corresponded to the wattage applied during the Åstrand test. As an example, we have a fictional subject aged 25 years with a body weight of 70 kg who has an estimated maximum heart rate of 208 beats per minute (bpm) – 0.7 x 25 = 191 bpm, a resting heart rate of 60 bpm, a heart rate of 90 bpm at 50 watts warming up, and a heart rate of 135 bpm at 175 watts Åstrand test which corresponds to an estimated VO_2_-max of 64.3 ml of O_2_/kg body weight/minute. Therefore, 30% of VO_2_-max corresponds to 19.3 ml of O_2_/kg body weight/minute. In this example, the relationship between ml of O_2_/minute and heart rate is

heartrate=2.15×(mlofO2/bodyweight/minute)+52.49

Therefore, 2.15 x 19.3 ml of O_2_/kg body weight/minute + 52.49 = 94 bpm at 30% of VO_2_-max. Next, the linear relationship between heart rate and wattage is

heartrate=0.36×watts+72

Therefore, solving the equation 94 bpm = 0.36 x watts + 72 returns 61 watts.

The oxidation of the ingested ^13^C-protein was exhaled as ^13^CO_2_. The CO_2_ production while resting was calculated by multiplying 300 mmol CO_2_/hour with the body surface area according to the Haycock formula [29]:

$$body\;surface\;area\;(m^{2})={weight\;(kg)}^{0.5378}\times {height\;(cm)}^{0.3964}\times 0.024265$$

The breath samples were measured for their ^13^CO_2_:^12^CO_2_ ratio and compared to a high ^13^C-enriched standard, i.e., Pee Dee Belemnite (PDB). The differences (delta, δ) between the breath samples and the standard is expressed as [30] follows:

$$\delta \;13C\;sample=\left(\frac{13C/12Csample}{13C/12Cstandard}-1\right)\times 1000$$

The PDB standard ^13^C/^12^C ratio is defined as 0‰. In order to calculate the ^13^C/^12^C ratio from the IRMS delta values, the following inversion formula was used [31]:

$$13C/12Cratio=\left((\frac{\delta \;13C\;sample}{1000})+1\right)*0.0112372$$

Next, the ^13^C/^12^C ratio of each breath sample was used to calculate the %^13^C [31] by

$$\%13C=\left(\frac{\frac{13C}{12Cratio}}{\frac{13C}{12Cratio}+1}\right)*100$$

The baseline (t = 0) breath sample ^13^C/^12^C ratio was subtracted from each subsequent breath sample to acquire the change from the subject’s baseline. The estimated CO_2_ production together with the δ ^13^C sample on each time point and the enrichment of the ^13^C-milk protein was used to calculate the exogenous protein oxidation rate of the subject at each time point with ten minutes in between. The change in ^13^CO_2_:^12^CO_2_ ratio over time has been described [32,33] by the general concentration model

$$y(t)=a\times t^{b}\times e^{-kt}+\varepsilon \;(normally\;distributed)$$

This function was fitted to the measurement data for each individual over time with *t* ranging from zero to 330 minutes by least squares estimation of the parameters *a*, *b*, and *k*. Total exogenous protein oxidation was calculated as the integral representing the area under the concentration curve (AUC). The error ε is assumed to have a normal distribution with zero mean and variance to be estimated from the residual error [34]. Each resulting curve begins at the subject’s natural amount, y(0) = 0. After ingestion at t = 0, the ^13^C-milk proteins are released by the stomach, digested, and become available via circulation to the cells for utilization or breakdown, including protein oxidation. The oxidation curve reflects the process of exogenous protein oxidation. The oxidation curve ascends from t = 0 onwards, reaches its maximum, and thereafter descends to the subject’s baseline over time.

The experimental design gives repeated measurements in time. Therefore, mixed models with random intercepts for the individuals were used to analyse the breath test measurements. For the statistical F-test for possible differences in mean between their habitual diet prior to each of the three breath measurements, a Type III Analysis of Variance with Satterthwaite’s method was employed on the following dietary outcomes: energy intake (kcal), protein intake (g/kg body weight/day), protein intake (g), en% protein (%), carbohydrates intake (g), en% carbohydrates (%), en% mono- and disaccharides (%), fat intake (g), en% fat (%), en% saturated fat (%), en% mono unsaturated fat (%), en% poly unsaturated fat (%), animal protein (g), and plant protein (g). The difference in mean total oxidation between the conditions within subjects was tested with the Satterthwaite's method F-test [35] by using random intercepts for them. Two other important characteristics directly calculated from the estimated parameters of each fitted curve y(t) per individual were the time point (t_max_) in minutes of when maximum oxidation rate was reached and the corresponding maximum value of the oxidation rate y(t_max_) where t_max_ = b/k. Its standard error per person was computed by the delta method [36].

All statistical analyses were performed by the statistical programming language R (R Core Team, 2019), using the library “nls” [37] for non-linear least squares [38] fitting of the concentration curves to the measurements over time and the delta method to determine the standard error of t_max_ [36]. All subjects outcomes per condition are statistically described as mean±SD.

## Results

Baseline characteristics of the sixteen male subjects are presented in Table 1. They exercised at 30% and 60% of their VO_2_-max and had an estimated mean of VO_2_-max of 56.2±9.7 ml O_2_/kg/min as determined by the Åstrand test. The World Health Organization has established 3.0–5.9 metabolic equivalent of task (MET) as moderate intensity and >6 METs as vigorous intensity [39] corresponding with moderate intensity exercise. The mean METs of exercising at 60% of VO2-max is equivalent to (0.6 x 56.2)/3.5 = 9.6 METs and is thus comparable to vigorous intensity exercise. The mean baseline ^13^CO_2_ enrichment of the individuals was not different between each experimental condition.

**Table 1:** Characteristics of the subjects (*n* = 16, unless otherwise specified).

	Mean	SD
**Age (years)**	23.8	4.0
**Height (cm)**	185	9
**Body weight (kg)**	78.7	9.5
**Body mass index (kg/m**^**2**^**)**	22.9	1.6
**Fat-free mass (%)**	87.2	4.9
**Habitual protein intake (g protein/kg bw/day)**	1.5	0.5
**Habitual protein intake (g protein/day)**	115	41
**Habitual En% protein (%)**	18	4
**Habitual En% carbohydrates (%)**	47	7
**Habitual En% fat (%)**	33	7
**VO**_**2**_**-max (ml/kg/min)**	56.2	9.7
**Moderate intensity exercise (watt)**	73	16
**Vigorous intensity exercise (watt)**	172	38
**Resting metabolic rate (kcal/day, *n* = 11)**	1880	211
**Baseline breath** ^**13**^**CO**_**2**_ **enrichment at rest (delta value)**	-25.08	0.69
**Baseline breath** ^**13**^**CO**_**2**_ **enrichment moderate exercise**	-25.13	0.39
**Baseline breath** ^**13**^**CO**_**2**_ **enrichment vigorous exercise**	-25.02	0.45

From the food diaries, the mean dietary intake of all of the subjects per condition is presented in Table 2. No significant differences in means were ascertained between experimental and baseline conditions for any of the dietary components. From the 24-hour urine collections, their mean (SD) characteristics urine are depicted in Table 2. No significant differences were determined between condition means for any of the urinary components.

**Table 2:** Characteristics of the subjects diet and 24-hour urine prior to each breath test (*n* = 16).

	Rest		30% exercise		60% exercise		*p-value*
	mean	SD	mean	SD	mean	SD	
**Energy intake (kcal)**	2557	639	2693	857	2733	799	0.336
**Protein intake (g/kg body weight/day)**	1.4	0.4	1.6	0.5	1.5	0.5	0.398
**Protein intake (g)**	109	30	122	44	115	48	0.350
** En% protein (%)**	18	3	18	3	17	4	0.388
**Carbohydrates intake (g)**	289	82	316	98	312	83	0.273
** En% carbohydrates (%)**	47	8	47	7	47	7	0.931
** En% mono- and disaccharides (%)**	18	6	18	8	18	7	0.872
**Fat intake (g)**	109	49	98	43	101	43	0.618
** En% fat (%)**	33	8	32	7	33	6	0.753
** En% saturated fat (%)**	12	4	11	3	12	4	0.308
** En% mono unsaturated fat (%)**	12	3	11	3	12	2	0.972
** En% poly unsaturated fat (%)**	7	2	7	2	6	3	0.685
**Animal protein (g)**	66	28	76	40	70	46	0.465
**Plant protein (g)**	43	11	42	13	44	9	0.889
**Urine (L/24-hour)**	1.84	0.89	1.89	0.64	1.79	0.75	0.896
** Urea (mmol/24-hour)**	469.0	175.6	474.2	180.9	464.5	149.2	0.948
** Creatinine (mmol/24-hour)**	16.9	5.3	17.3	5.6	16.2	4.0	0.503

All of the subjects completed the rest and exercise conditions as well as subsequent breath test measurements. After curve fitting of the concentration function y(t) = a× t^b^×e^-kt^ for each of them and their condition, the overall mean of constant *a* is 0.019±0.039. For constant *b*, the overall mean is 2.247±0.832. For the constant *k*, the overall mean is 0.018±0.008. The concentration curve fitted well to all breath test measurements according to *R*^2^ 0.926±0.044. The *R*^2^ proportion of variance explained per breath test condition was for rest 0.910±0.046, for moderate intensity exercise 0.920±0.051 and 0.947±0.021 for vigorous intensity exercise. Mean residual standard deviation of all of the measurements to the fitted curve was 0.685±0.213.

**Fig 1:**
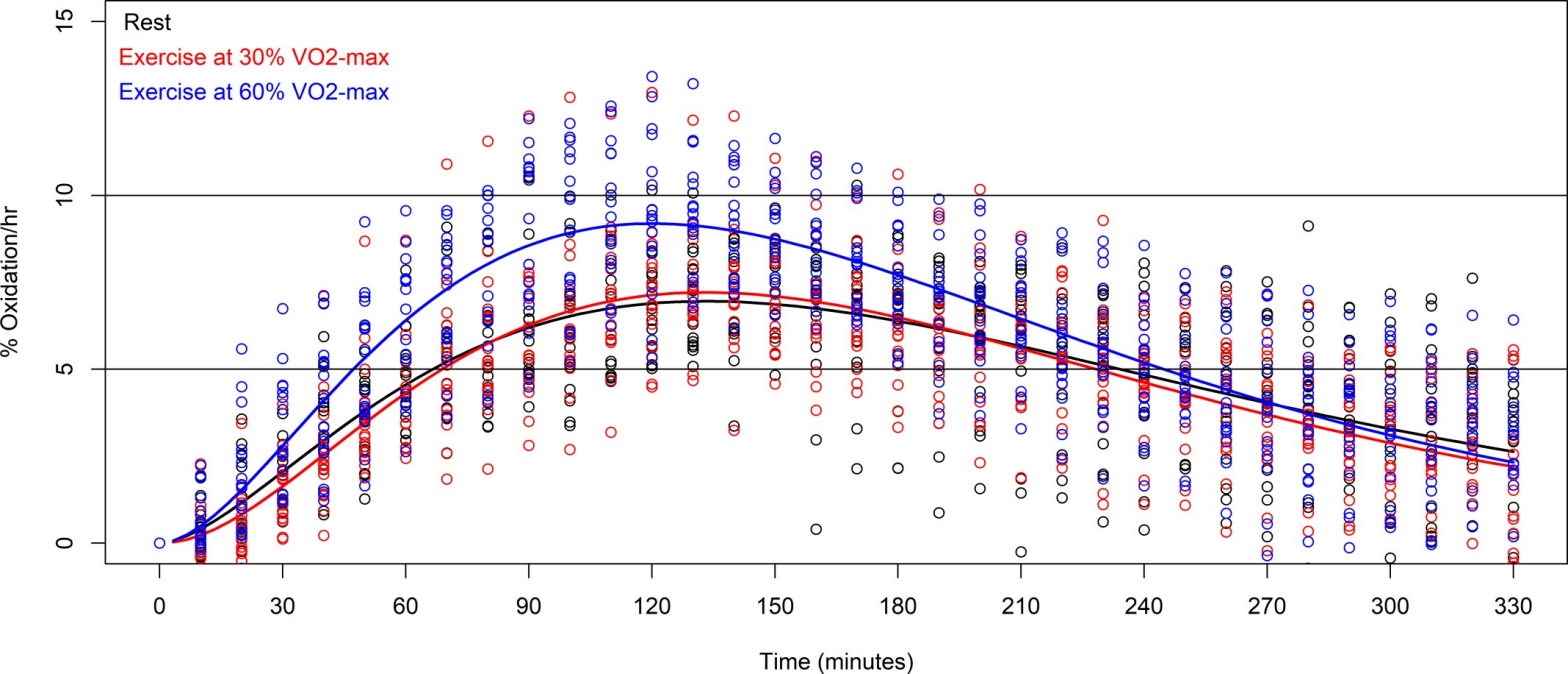
Exogenous protein oxidation kinetics during resting (black), after exercise at 30% VO_2_-max (red), and after exercise at 60% VO_2_-max (blue)(*n* = 16).

**Fig 2:**
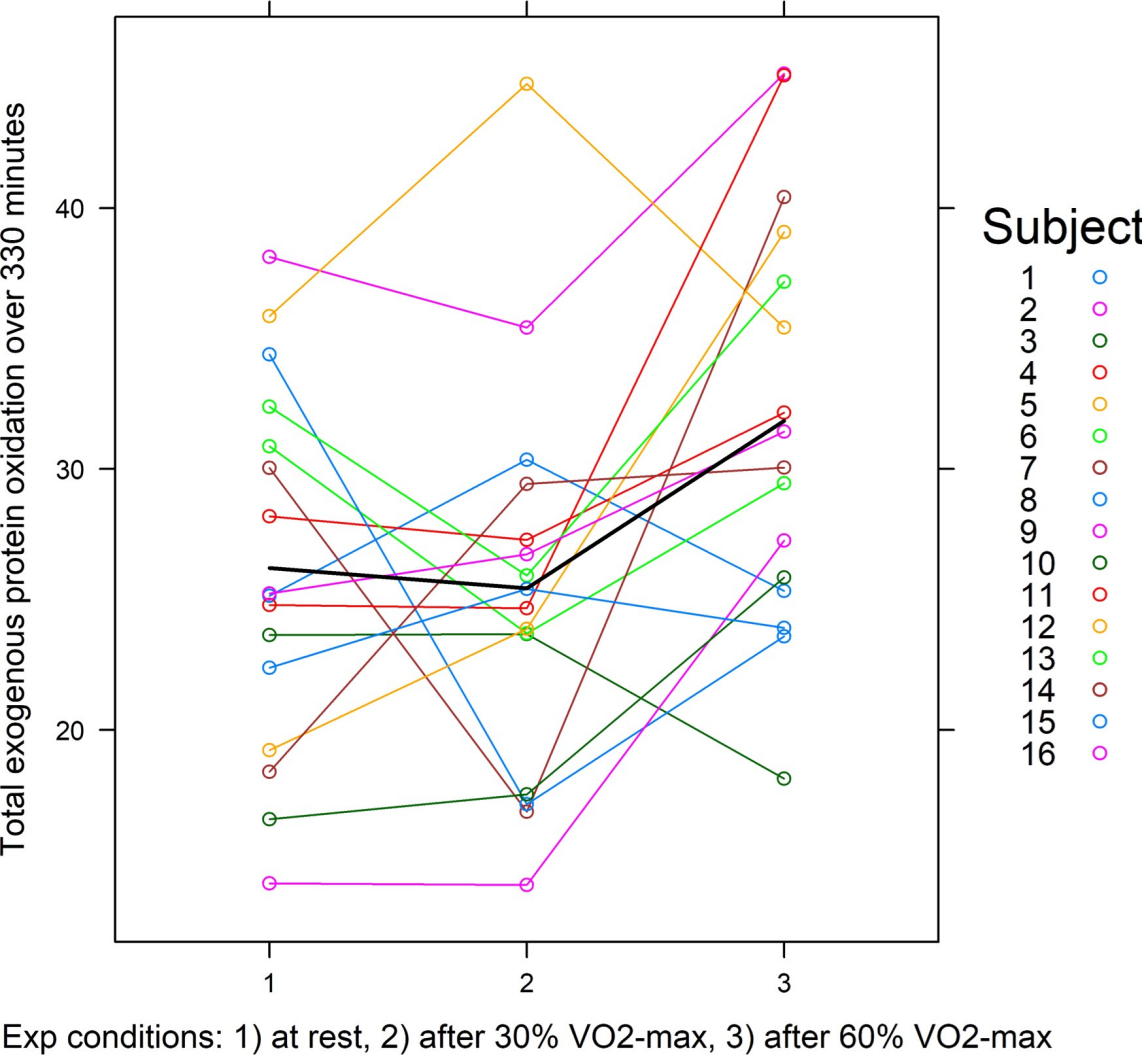
Total exogenous protein oxidation over 330 minutes as measured by the area under the concentration curve per subject at rest and after two levels of exercise (15 minutes cycling at 30% and 60% of subject’s VO_2_-max). The mean is represented by the black line.

The average exogenous protein oxidation kinetics of all of the subjects during the 330 minutes breath test, for each of the conditions resting, exercise at 30%, and exercise at 60% are shown in Fig 1. Total exogenous protein oxidation (AUC) after resting was 26.2±7.1%, after exercise at 30% was 25.4±7.6%, and after exercise at 60% was 31.8±8.0%. The difference in mean total oxidation between resting and exercise at 30% was not statistically significant (*p* = 0.711, *t* = -0.374, *df* = 30). The variance in mean total oxidation between resting and exercise at 60% was statistically significant (*p* = 0.012, *t* = 2.681, *df* = 30). The total exogenous protein oxidation (AUC) per subject over the breath tests are shown in Fig 2.

The average *t*_max_ time in the resting condition was 142±37 minutes, for exercise at 30%, *t*_max_ was 137±21 minutes, and for exercise at 60%, *t*_max_ was 123±16 minutes. The difference in mean *t*_max_ between resting and exercise at 30% was not statistically significant (*p* = 0.454, *t* = -0.758, *df* = 30) and that between resting and exercise at 60% was statistically significant (*p* = 0.013, *t* = -2.634, *df* = 30).

The mean maximum %oxidation rate per hour after resting was 7.5±1.2%, after exercise at 30% was 7.5±1.7%, and after exercise at 60% was 9.4±1.7%. The difference in mean maximum %oxidation rate per hour between resting and exercise at 30% was not statistically significant (*p* = 0.981, *t* = 0.024, *df* = 30). The difference in mean maximum %oxidation rate per hour between resting and exercise at 60% was statistically significant (*p*<0.001, *t* = 4.876, *df* = 30).

## Discussion

In the current study, we found that exogenous protein oxidation increases immediately after vigorous intensity exercise but not after moderate intensity exercise compared to the resting condition.

Our finding that ingestion of 30 g of ^13^C-milk protein led to total exogenous oxidation of ~25% after the resting condition, corresponding to ~7.5 g, is in accordance with our previous studies wherein the mean total exogenous oxidation under equivalent conditions was ~7.5 g [40] and ~9.0 g, respectively [34]. The equivalent conditions consisted of healthy male subjects consuming their habitual diet. Then after an overnight fast, the subjects ingested 30 g of ^13^C-milk protein and were subsequently measured for their exogenous oxidation in the rested condition over 330 min. Likewise, our findings are also in accordance with a study in which after resistance exercise, the subjects received a primed constant infusion of [1-13C]leucine and ingested different doses of whole egg protein to measure the protein synthesis response [41]. In the latter study, it was determined that 20 g of protein resulted in a maximal protein synthesis response and that dietary protein in excess (40 g) stimulated irreversible oxidation [41]. Extrapolating this finding to our study suggests that the ingestion of 30 g of ^13^C-milk protein leads to a total oxidation of ~10 g which is in the same order of magnitude as the current finding of ~7.5 g. The absence of a change in exogenous protein oxidation induced by moderate intensity exercise concurs with studies measuring glucose and fat metabolism [12,42]. The metabolic responses during and after moderate intensity exercise remain relatively stable as reflected by substrate availability (i.e., glucose and free fatty acids) and oxidation, although the metabolic response changes with increasing intensity [12,42]. We observed a comparable difference between exogenous protein oxidation after moderate intensity, i.e., no change, and after vigorous intensity exercise, i.e., increase in exogenous protein oxidation. This suggests the existence of an exercise intensity threshold above which post-exercise exogenous protein oxidation increases.

We showed that exogenous protein oxidation significantly increases after vigorous intensity exercise which suggests a decrease in the utilization of ingested protein and that this pace of exercise is a metabolic stressor. This is in contrast to literature in which especially resistance exercise but also aerobic exercise was found to be an important stimulus for post-exercise protein synthesis, increased protein turnover rate and, consequently, protein utilization [12–15]. For example, the myofibrillar protein synthesis is increased after both moderate and vigorous intensity aerobic exercise between 0.5–4.5 hours [15]. Another study performed in young and older men who walked on a treadmill for 45 minutes at 40% of VO_2_-peak found that both muscle protein synthesis and breakdown were acutely increased, indicating an increased muscle protein turnover rate [43]. It was reasoned that the increased turnover rate was likely caused by the cellular repair and maintenance response [43]. The apparent discrepancy between both increased exogenous protein oxidation and increased protein synthesis after exercise can be solved if these processes occur at different time frames and/or with different levels of activity, resulting in net protein synthesis. This concept is, in part, supported by the fact that aerobic exercise does not lead to significant muscle mass depletion [44].

Although this study was not designed to unravel the underlying causal mechanisms of exogenous protein oxidation, two possible explanations for our finding seem worthwhile to consider. First, protein oxidation releases energy, therefore, the increase in protein oxidation after vigorous intensity exercise could act as a response to partially meet an increased energy demand. Second, this pace of exercise induced an increase in protein turnover rate which led to the increased post-exercise protein oxidation. With regard to the first possible explanation, a positive linear relationship between leucine oxidation and exercise intensity has been found, although the relative contribution of protein to fuel metabolism decreases with increasing energy demands [45,46]. In our study, the mean increase in exogenous protein oxidation after vigorous intensity exercise compared to resting was 1.68 g (5.6% of 30 g). The caloric content of 1.68 g protein equates to 6.72 kcal (1.68 g × 4 kcal/g protein) and could, for example, sustain resting energy demands for 5.6 minutes in an individual with a resting metabolic rate of 1750 kcal/day. This amount of energy is relatively small with respect to resting metabolic rate and, therefore, it is unlikely that the reaction of the increase in exogenous protein oxidation was instigated to (partially) fulfill the increased post-exercise energy demand.

The second possible explanation for the increase in post-exercise exogenous protein oxidation after vigorous intensity exercise is based on the demonstrated strong correlation between the metabolic rate and leucine flux [47]. The latter is an index of protein degradation [47] which is subsequently part of the protein turnover process. An increased metabolic rate after vigorous intensity exercise is plausible and, therefore, would increase the protein turnover rate. The protein turnover process is ~90% efficient in recycling degraded proteins derived amino acids [16]. The remaining ~10% of amino acids are unavoidable losses and thus oxidized [16]. Therefore, when the protein turnover rate increases, protein oxidation also increases.

The design of our study allows us to make qualitative statements on the relative changes in exogenous protein oxidation under different physiological circumstances, however it is important to acknowledge the limitations surrounding the quantitative statements made in the discussion earlier. The strength of the quantitative statements is limited because first, we did not assess the impact of the bicarbonate pool. Second, the CO_2_ production was estimated instead of measured, which in principle makes the quantitative oxidation an estimation. Finally, we did not include a exercise control measurement to assess the probably small effect of changes in background ^13^CO_2_ production. We have implemented overnight fasting in order to achieve similar metabolic starting conditions in our participants. The fasting period ended after the rest and exercise conditions with the ingestion of the ^13^C-milk protein test drink. Therefore, the exercise-induced increase in energy demand could only be satisfied by releasing energy from body energy stores. As a result, a limitation of this study is, that it cannot be ruled out that the subjects were partially carbohydrate depleted post-exercise which could have influenced subsequent exogenous protein oxidation [48]. Another possible limitation of our study is, that variances in habitual diet components such as different levels of protein and energy intake can influence protein metabolism and thus also affect protein oxidation[49,50]. In our study, we did not standardize the participants’ habitual diets which could have led to difficulties in interpreting the results. However, the mean composition of their diets taken from the food diaries were not statistically different between the breath test conditions. Similarly, the urea and creatinine excretion, measured with the 24-hour collections, were also not statistically varied between the different breath test conditions. Therefore, it seems unlikely that the participant’s habitual diets confounded the effect of exercise on exogenous protein oxidation.

The role of exogenous protein oxidation has two possible implications for anabolic competence. Initially, measuring an increase in exogenous protein oxidation appears to solely suggest a loss of protein which seems to conflict with the goal of lean body mass maintenance. However, as exercise stimulates changes in skeletal protein metabolism, which is reflected by an increased protein turnover rate [50], a concomitant increase in protein oxidation is to be expected. For the future, it is necessary to differentiate between an increase in exogenous protein oxidation representing a healthy response geared towards anabolism or an excessive response leading to catabolism. To address this issue, future experiments should measure exogenous protein oxidation in parallel with the measurement of protein turnover rate in healthy individuals and patients with disturbed protein metabolism under resting conditions.

In conclusion, exogenous protein oxidation increases immediately after vigorous intensity exercise but not after moderate intensity exercise compared to the resting condition. This suggests the existence of an exercise intensity threshold above which post-exercise exogenous protein oxidation increases. Regarding the paradigm of anabolic competence, the results suggest that vigorous intensity exercise itself is a metabolic stressor as reflected by immediate post-exercise increase in exogenous protein oxidation, although exercise leads to conservation of lean body mass with sufficient recovery time.

## Supporting information

S1 Table Primary data of the study. (XLSX)
